# A retrospective audit of audiology encounters in patients undergoing Cisplatin treatment at a large Australian tertiary cancer care centre

**DOI:** 10.1007/s11764-024-01689-x

**Published:** 2024-10-15

**Authors:** Georgia M. Lester, Wayne J. Wilson, Barbra H. B. Timmer, Rahul M. Ladwa

**Affiliations:** 1 Discipline of Audiology, School of Health and Rehabilitation Sciences, The University of Queensland, Brisbane, 4072 Australia; 2 Sonova AG, Staefa, Switzerland; 3 Princess Alexandra Hospital, Metro South Hospital and Health Service, Queensland Health, Brisbane, Australia; 4 Faculty of Medicine, The University of Queensland, Brisbane, Australia

**Keywords:** Ototoxicity, Hearing loss, Audiology, Chemotherapy, Hearing monitoring, Quality of life

## Abstract

**Purpose:**

To identify the number and timing of audiology encounters for adult oncology patients in a tertiary care setting in Australia.

**Setting (population):**

A retrospective case review was completed for 149 patients who received Cisplatin chemotherapy (CT) at a large, publicly funded tertiary hospital in Brisbane, Queensland, Australia between 1st January and 31st December 2019. Patient data was extracted from the Queensland Oncology Repository (QOR) provided by Cancer Alliance Queensland (CAQ).

**Results:**

The number of audiology encounters was low overall with a median of 0 and interquartile range (IQR) of 0–1. Of the entire patient cohort, there was a mean of 1.2 encounters with 56% of patients not engaging with audiology. Where audiology did occur, encounters were most likely before or early in the CT treatment period.

**Conclusions:**

This study has demonstrated engagement with audiology services for patients undergoing CT treatment was limited with the few audiology engagements occurring before or early in the CT treatment period. Further research is needed to identify the barriers and facilitators to accessing audiological ototoxic monitoring (OtoM) during chemotherapy treatment in hospitals in Australia.

**Implications for cancer survivors:**

Early identification of ototoxic hearing loss offers the opportunity to minimise further exposure to the ototoxic agent, minimise functional and communication impacts for the patient and provide early opportunity for discussion, education and counselling with patients, carers and their treating team. This, in turn, is expected to improve health related quality of life.

**Supplementary Information:**

The online version contains supplementary material available at 10.1007/s11764-024-01689-x.

## Introduction

For individuals undergoing chemotherapy treatment for a cancer diagnosis, health care needs can be high and complex. The dynamic nature of these diagnoses, related symptoms and side effects demands an experienced multidisciplinary team (MDT) comprising specialist physicians and allied health (AH) professionals including audiologists, pharmacists, speech pathologists, dietitians, psychologists, social workers, occupational therapists and physiotherapists. Whilst each AH profession involved in cancer care plays a role in managing treatment side effects and endeavouring to preserve quality of life (QoL), some AH professionals are more heavily utilised than others at distinct intervals during the acute phases of chemotherapy treatment.

In Australia, the primary role of audiologists working with oncology patients in hospital settings is to complete audiological ototoxicity monitoring (OtoM) (with patients referred to audiology services outside of the hospital system for complex rehabilitation or intervention, if needed). Ototoxicity monitoring sees the audiologist regularly assess patient hearing (and possibly balance) for signs of ototoxicity, where ototoxicity is the damage to the auditory and/or vestibular structures of the inner ear caused by ototoxic agents. Whilst ototoxic effects can include tinnitus and vertigo in a range of patients, this paper focuses on the ototoxic effect of hearing loss seen in 50–80% of oncology patients receiving platinum-based chemotherapy [1]. To manage this high level of risk, audiology groups in the United States of America (USA) [2, 3] and South Africa [4] have recommended patients undergoing platinum-based chemotherapy receive audiological OtoM in the form of regular behavioural tests of hearing thresholds and objective measures of cochlea hair cell function [5]. Such OtoM typically involves a pre-treatment baseline assessment and regular, repeated testing with a goal of identifying any shifts from baseline. Completing audiological OtoM is seen as an opportunity for discussion, education and counselling with patients, carers and their treating team to minimise further exposure to the ototoxic agent and its functional and communication impacts for the patient.

Though research in the area is limited, current literature suggests poor uptake and effectiveness of audiological OtoM for oncology patients during chemotherapy. Existing publications from countries including the USA, Canada, New Zealand, the United Kingdom (UK) and South Africa cite reasons for this including inconsistent protocols for OtoM and inconsistent referrals to audiology [5], unclear referral pathways for physicians [6], environmental noise whilst testing in a hospital ward, the compromised health status of patients [7], poor awareness of OtoM procedures [7, 8], the lack of involvement and consultation with audiologists through the initial phases of evaluation and treatment discussions [9], and scheduling limitations, physical resourcing and staffing issues in audiology [10]. The extent to which patient demographic variables and diagnoses affect engagement with AH services including audiology is not yet known. Also unknown is the chronology of encounters and context in which audiology and other AH engagement occurs. To the authors’ knowledge, the present study is the first to investigate audiological OtoM practices, barriers and facilitators by mapping audiology and other AH encounters for oncology patients receiving platinum-based chemotherapy in a hospital setting.

The primary aim of this study was to identify the number of audiology encounters and map where these encounters occurred during treatment in a sample of adult oncology patients who received at least one dose of CT at a large publicly funded tertiary hospital in Brisbane, Queensland, Australia between 1st January and 31st December 2019. The secondary aim was to identify and map the number of other AH encounters in the same sample of adult oncology patients for comparison against their audiology encounters.

## Patients and methods

### Research design

A retrospective analysis of oncology and AH encounters for patients aged 16 and over who received at least one dose of ototoxic CT in the cancer services of a large publicly funded tertiary hospital in Brisbane, Queensland, Australia. As the Covid-19 pandemic caused major interruptions and changes to the delivery of medical and AH services in Queensland from the beginning of 2020, the window of data used in this study was limited to participants who commenced CT treatment between 1st of January and 31st of December, 2019. Any medical and AH encounters that occurred up to 30 days preceding and 365 days following this CT start date were included. This recruitment window was chosen to provide insight into the typical number and pattern of patient encounters prior to the onset of Covid-19 service disruptions whilst also considering any preceding AH engagement and the time required for patients to complete any encounters associated with that acute course of treatment. Patients who received CT specifically were targeted as the population of interest as CT is one of the most effective, used and known ototoxic medication in cancer treatment [11].

### Patients

Participant data was drawn from the Cancer Alliance Queensland’s (CAQ), Queensland Oncology Repository (QOR), which is a centralised repository holding information collected from over 60 data systems and cancer care providers across Queensland [12]. The dataset analysed contained 149 adult patients with a cancer diagnosis who received at least one dose of CT based chemotherapy during the study period. Nine unique patients were removed from the original data set as they received CT but did not have a cancer diagnosis recorded. One additional patient was removed as they had CT start and end dates but no actual CT encounter was recorded.

### Facility

This study was conducted using data from a publicly funded tertiary hospital where there is no fee for patients to access inpatient or outpatient medical or AH services. The metropolitan region of Brisbane in which the study site is located is culturally and linguistically diverse with the hospital providing cultural support, interpreter services and financial subsidies for patients as required [13]. The site is a tertiary referral centre supporting patients with an array of cancer diagnoses [14] with its patient caseload representative of the general population of cancer patients who would access AH services in a public health facility in Australia. The hospital includes various on-site AH departments including audiology.

### Data collection

Deidentified data regarding patient demographics, diagnoses and hospital encounters were collected to obtain insight into patient engagement with audiology and other AH services in the period surrounding CT treatment. The full list of data provided for this study can be seen in the online resources and included patient demographic (age, sex, socio-economic status and location) and diagnoses information (primary site and morphology) as well as the date and type of all medical and AH encounters that occurred during the study period. The AH encounters recorded included audiology as well as pharmacy, speech pathology, dietetics, psychology, social work, occupational therapy, physiotherapy and podiatry. Of note, the data provided on patient encounters included only encounters that were coded by the hospital as having occurred, not those that may have been requested by physicians, booked and/or cancelled, those that were not attended or patients who may still be on a wait list. From the data provided there was no way to ascertain the relationship between AH encounter and CT treatment (whether the encounter was related to the patient’s cancer diagnosis and treatment or was unrelated entirely). This is understood to be a major limitation of the data. Whilst all diagnoses, patient-reported side effects, procedures, consultations and other medical encounters that occurred during the study period were provided in the original data set, only AH encounters and encounters coded as the administration of CT were of interest and included in the analysis.

Prior to data analysis, some manual recoding was performed to recategorize variables for statistical analysis. Comprehensive details on the primary site of malignancy were originally provided per the International Statistical Classification of Diseases and Related Health Problems, Tenth Revision, Australian Modification (ICD-10-AM). These primary sites were recoded into one of two categories based on the physical location of the primary site with ‘head and neck’ being either systemic or with a primary site located in the head and neck region, and ‘other’ being the site of malignancy anywhere else on the body. Amongst other considerations, site was stratified as a variable of interest due to the concurrent use of Cisplatin with radiation in head and neck cancer patients [15] and potential bias and prioritisation of audiology and OtoM when the site is physically located closer to the ear and cochlea. Age was recorded as the age of the patient at the time of their initial CT treatment. Both socio-economic status (SES) and location (the patient’s usual residence at the time of their treatment) were analysed as ordinal variables and were included in the analysis as possible determinants of accessibility to the service (e.g., was attendance impacted by transport issues or travel time). The Socio-Economic Indexes For Areas (SEIFA) 2021 was used to calculate the categories of SES with deciles 1–2 categorised as disadvantaged, 3–8 categorised as middle and 9–10 categorised as affluent. Patients were recorded as ‘survived’ if they were alive at the end of study period. To create the timeline of patient encounters, a plot of all encounters against days in the study period was created for each profession and each encounter was manually coded as having occurred in one or more of 5 periods: before, early (in the first third of the treatment period), middle (in the middle third of the treatment period), late (in the last third of the treatment period) and after CT treatment.

### Data analysis

Data analysis for this study was completed in four stages. First, descriptive statistics were used to summarise and describe the basic demographic and diagnostic information of patients included in data set. Second, all patient encounters with AH services were quantified as the total number of encounters for each patient, the number of AH encounters for each patient, the total number of encounters for all patients for each profession, the number of CT chemotherapy treatments in total and the number of CT chemotherapy treatments for each patient. Third, quantitative data regarding patient encounters and demographic information were investigated for correlations using Spearman’s correlation coefficient and differences using a two-tailed Mann–Whitney *U* test at the 1% significance level to mitigate for the use of multiple comparisons. Fourth, a heat map of patient encounters was created to assess the concentration of audiology and AH engagement at each of the five periods of CT treatment.

### Ethics approval

Ethics approval was received from Metro South Human Research Ethics Committee (HREC)(HREC/2022/QMS/99194) and the University of Queensland (2022/HE002149) prior to commencement of the study. All patients consented to the use of their deidentified data for research purposes at the time of their treatment.

## Results

Table 1 shows patient demographic and diagnostic information. Initial analyses showed that the study demographic was predominantly older, middle-class males with a primary site of cancer diagnosis being head and neck.

**Table 1:** Sociodemographic characteristics of patients who received CT at the study site during the study period

		*n* = 149	%
Age	16–35	9	6
	36–65	44	30
	> 65	96	64
Sex	Male	109	73
	Female	40	27
Survived	Yes	117	78
	No	32	22
Location	Major city	113	76
	Inner regional	16	11
	Outer regional	20	13
SES	Affluent	15	10
	Middle	92	62
	Disadvantaged	42	28
Primary site	Head and neck	96	64
	Other	53	36

**Fig. 1:**
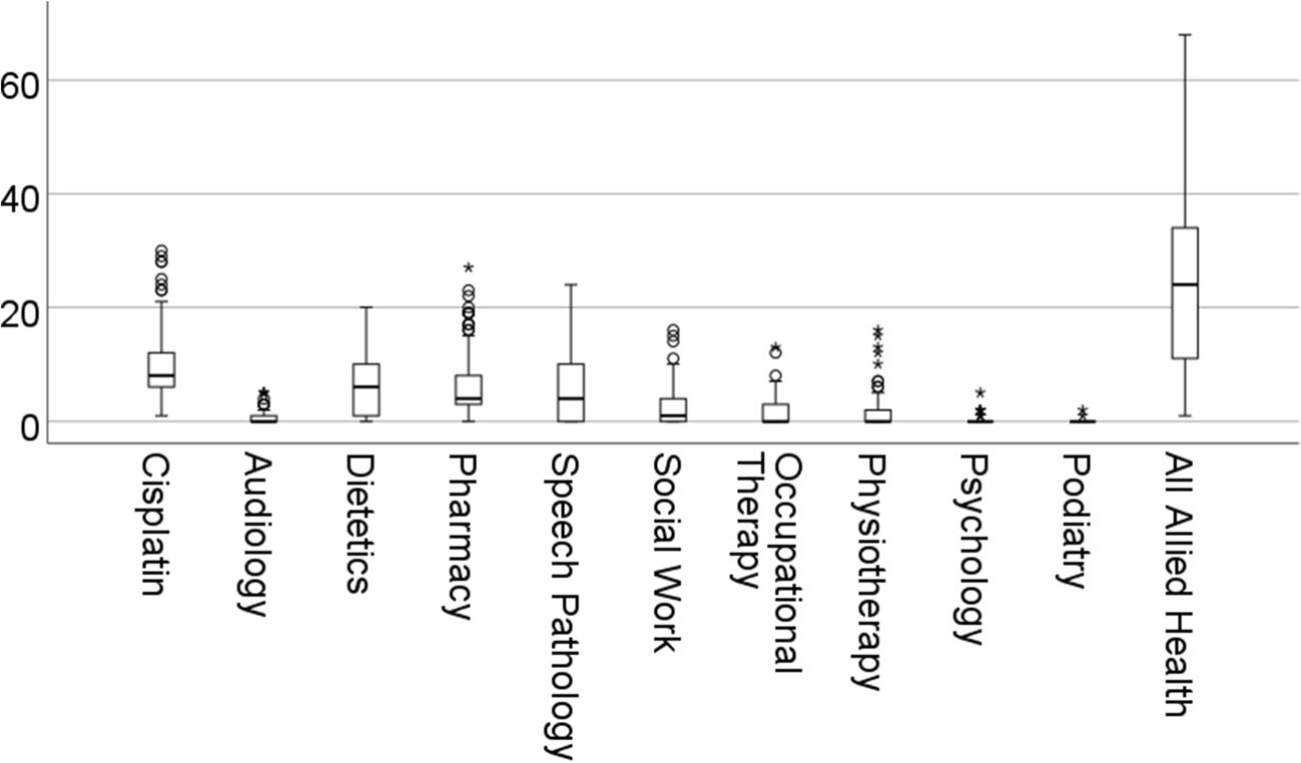
Box and whisker plots of the total number of CT, audiology and AH service encounters recorded during the study period (bold horizontal lines = median values, boxes = interquartile ranges (IQR), whiskers = minimum and maximum values [less outliers], and circles and asterisks = outliers > 1.5 and > 3 (respectively) × IQR above the 75.^th^ percentile)

Figure 1 shows the number of audiology and other AH visits relative to CT treatments. The number of audiology encounters was very low relative to other AH encounters, with the exceptions of psychology and podiatry.

Table 2 shows that patients with a primary malignancy in the head and neck area were significantly more likely to receive audiology than those with primary sites in any other area of the body (*z* =  − 3.456, *p* =  < 0.001), as were those who survived the study period (*z* =  − 3.139, *p* = 0.002) compared to those who did not. There was no significant relationship between the number of audiology encounters and the number of CT treatments or patient location, socioeconomic status or sex.

**Table 2:** Relationships between number of patient encounters with CT, audiology and other AH services and patient diagnostic and demographic variables

	Spearman’s correlation (*r*)				Mann–Whitney *U* (*z* score)		
	Age	Location	SES	CT	Sex	Site	Mortality
CT	− 0.248*	0.109	0.033	-	− 0.088	− 3.326*	− 1.125
Audiology	− 0.159	− 0.004	− 0.101	− 0.009	− 1.309	− 3.456*	− 3.139*
Speech pathology	0.208	− 0.015	0.057	− 0.222*	− 2.835*	− 8.846*	− 3.289*
Dietetics	0.168	− 0.009	0.043	− 0.129	− 2.389	7.595*	− 3.252*
Physio	0.038	0.010	− 0.111	0.001	− 1.443	− 0.239	− 0.047
Occupational therapy	0.019	− 0.082	0.004	− 0.025	− 0.799	− 2.883*	− 1.329
Social work	− 0.097	− 0.193	0.038	0.032	− 0.684	− 3.740*	− 1.389
Podiatry	0.044	0.065	0.127	− 0.022	− 0.860	− 0.418	− 0.974
Psychology	− 0.218*	0.098	− 0.045	0.110	− 0.188	− 2.345*	− 0.895
Pharmacy	− 0.101	0.152	− 0.023	0.494*	− 1.285	− 4.665*	− 2.312
All AH	0.056	0.024	0.040	0.066	− 1.403	− 5.361*	− 2.957*

Note: *p < 0.01

Table 3 shows the concentration of audiology and other AH encounters by period of CT treatment. The highest concentration of audiology encounters occurred before patients commenced any CT treatment. The highest concentration of encounters overall was for pharmacy, particularly in the early periods of CT. Other AH professions showed varying patterns of patient engagement with some remaining relatively consistent throughout the entire course of treatment (dietetics, speech pathology, physiotherapy) and others showing concentrations of encounters before and/or early (social work) and late and/or after (occupational therapy) CT treatment.

**Table 3:**
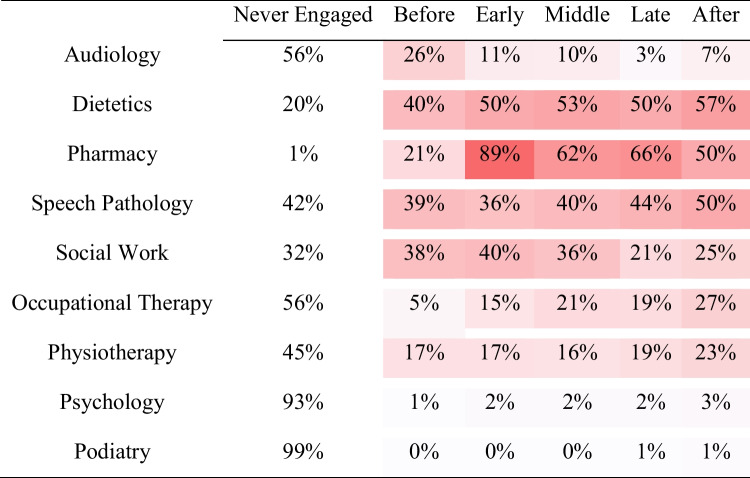
Heatmap of encounters with the percentage of patients who engaged with audiology and other AH professionals in each of the periods of CT treatment

The heatmap in Table 3 uses increasing colour intensity to map increasing concentrations of encounters across all periods of CT treatment and allied health professions

## Discussion

The number of audiology encounters experienced by the participants during the study period was low with a median of 0 and interquartile range (IQR) of 0–1. Of the entire patient cohort, there was a mean of 1.2 encounters with 56% of patients never engaging with audiology. This finding was unexpected due to the hospital study site having an on-site audiology department and the presence of international guidelines recommending audiological OtoM occur during CT treatment. Inspection of the results presented in Table 2 offers some insights into factors that could have influenced the number of audiology encounters. The number of audiology encounters was not affected by participant demographics (location, SES and sex) or the number of CT treatments (although these findings must be considered within the larger finding of a median of 0 participant encounters with audiology). This consideration suggests that the larger problem of low participant encounters with audiology is less likely to do with the demographic and diagnostic factors considered here, and is more likely to do with hospital and systemic processes relating to care and referral coordination, audiology workload and absence of audiological OtoM protocols for audiologists in the hospital. This conclusion is consistent with similar reports published by multiple authors [5–10] and is supported by the distribution of audiology encounters in Table 3 that suggests patients who attended an initial baseline assessment before commencing CT treatment were rarely then engaged for monitoring or review.

Compared to audiology, participant encounters with other AH professions ranged from higher to lower during the treatment period suggesting that different priorities had been assigned to different AH supports during a patients CT treatment. Compared to audiology encounters to monitor for potential hearing changes, the substantially higher numbers and broader spread of encounters with speech pathology and dietetics suggest that a higher priority was given to managing known complications of head and neck cancers such as mucositis (inflammation), xerostomia (dry mouth) and dysphagia (difficulty swallowing) [16] and any side effects resulting malnutrition, dehydration and weight loss [17–19]. Whilst AH input for nutrition and swallowing complications was expected to be prioritised, the lower number of encounters with psychology was somewhat surprising given the significant and far-reaching impacts of cancer on patient quality of life and psychological well-being [20] with head and neck cancers being particularly distressing and traumatic [21–23]. Whilst postulative, the higher number of social work encounters could suggest the socio-emotional supports needed by some participants were met by social work without the need for referral to clinical psychology.

## Limitations

The present study had at least three limitations. First was the data being limited to records of encounters only, with no details of the reason for referral, wait time or outcome. Second was the data being limited to a single, tertiary, public hospital facility in Brisbane, Queensland, Australia. Whilst the population served by this hospital is culturally and demographically diverse, the sampled participants could differ from populations served by other hospitals in Australia. The data sample also excluded any referrals that were made to external services including audiology in the community. Third was the absence from the dataset of variables that could have influenced participant encounters such as work status, access to transport, marital status and carer responsibilities.

## Future research

Future research should investigate reasons why audiology encounters might be so low for patients receiving CT treatment in large public hospitals in Australia and why the concentration of encounters is present only in the beginning and early period of treatment. This lack of continuity is of particular interest as existing OtoM guidelines recommend regular and ongoing monitoring for patients undergoing ototoxic CT. Future investigations should consider the patients journey through their CT treatment to identify both the nature and the timing of any barriers preventing patients from accessing adequate audiological care. Future research would also do well to survey the thoughts and opinions of audiologists, medical and other AH professionals working in the area.

## Conclusion

This study has demonstrated engagement with audiology services for patients undergoing CT treatment was limited with the few audiology engagements recorded occurring before or early in the CT treatment period. Such poor engagement with audiology could deny patients access to the information needed to make informed decisions regarding their hearing during their chemotherapy treatment. Further research is needed to identify the barriers and facilitators to accessing audiological OtoM and other AH support during CT treatment in hospitals in Australia.

## Supplementary Information

Below is the link to the electronic supplementary material.

Supplementary file1 (DOCX 16 KB)

Supplementary file2 (DOCX 16 KB)

Supplementary file3 (DOCX 17 KB)

## Data Availability

The data analysed in this study is available from the corresponding author upon reasonable request.
