# Supplementary material for: A retrospective audit of audiology encounters in patients undergoing Cisplatin treatment at a large Australian tertiary cancer care centre
Source: J Cancer Surviv. 2024 Oct 15;20(2):816–22. doi: 10.1007/s11764-024-01689-x (PMC12988975; doi:10.1007/s11764-024-01689-x)
Supplement: Supplementary file 1 — Supplementary file1 (DOCX 16 KB) [file 11764_2024_1689_MOESM1_ESM.docx]

**A retrospective audit of audiology encounters in patients undergoing Cisplatin treatment at a large Australian tertiary cancer care centre**

Georgia M. Lester, Wayne J. Wilson, Barbra H. B. Timmer and Rahul Ladwa

Corresponding author email: [georgia.lester@uq.edu.au](mailto:georgia.lester@uq.edu.au).

Supplementary Material 1: List of data points and descriptions provided by Cancer Alliance Queensland to the research team

| Field Name | Field Description |
| --- | --- |
| Studyid | Study id |
| Sex | Male/Female |
| Date of birth | Date of birth |
| DiagRemoteness_Gpr1 | ASGS ABS classification based on SA2 of residence at diagnosis https://www.abs.gov.au/statistics/statistical-geography/australian-statistical-geography-standard-asgs |
| DiagRemoteness_Gpr2 | ASGS ABS classification based on SA2 of residence at diagnosis |
| DiagRemoteness_Gpr3 | ASGS ABS classification based on SA2 of residence at diagnosis |
| DiagSESDecile | Socioeconomic classification is based on the Socio-Economic Indexes for Areas (SEIFA), a census-based measure of social and economic well-being developed by the Australian Bureau of Statistics (ABS) and aggregated at the level of Statistical Areas Level 2 (SA2s)- deciles https://www.abs.gov.au/websitedbs/censushome.nsf/home/seifa |
| DiagSESName | Socioeconomic classification is based on the Socio-Economic Indexes for Areas (SEIFA), a census-based measure of social and economic well-being developed by the Australian Bureau of Statistics (ABS) and aggregated at the level of Statistical Areas Level 2 (SA2s) |
| HasCancerDiagnosis | Has cancer diagnosis |
| Diagnosis date | Date of cancer diagnosis |
| DiagPrimarySiteCode | Primary site (ICD-10-AM 12th edition) https://ace.ihacpa.gov.au/ICD10AMTwelfthEdition.aspx |
| DiagPrimarySiteName | Primary site description |
| DiagMorphologyCode | Morphology (ICD-10-AM 12th edition) |
| DiagMorphologyName | Morphology description |
| HadCisplatin | Protocol LIKE '%cisplatin%' OR DrugName = 'Cisplatin' |
| CTStartDate | Cisplatin start date |
| CTEndDate | Cisplatin end date |
| HasNAPServiceEvent | Had Non admitted service event https://www.health.qld.gov.au/hsu/collections/qhnapdc |
| NAPServiceEventDate | Non admitted service event date |
| NAPServiceRequestDate | Non admitted service request date |
| NAPLocalClinicCode | Non admitted service event local clinic code (PAH Cent, PAH Caudio, PAH CSECU) |
| NAPCorporateClinicCode | Non admitted service event corporate clinic code |
| NAPCorporateClinicName | Non admitted service event corporate clinic name |
| EventStartDate | Non admitted, admitted or RT start event date |
| EventEndDate | Admitted discharge date or RT end date |
