# Supplementary material for: A retrospective audit of audiology encounters in patients undergoing Cisplatin treatment at a large Australian tertiary cancer care centre
Source: J Cancer Surviv. 2024 Oct 15;20(2):816–22. doi: 10.1007/s11764-024-01689-x (PMC12988975; doi:10.1007/s11764-024-01689-x)
Supplement: Supplementary file 2 — Supplementary file2 (DOCX 16 KB) [file 11764_2024_1689_MOESM2_ESM.docx]

**A retrospective audit of audiology encounters in patients undergoing Cisplatin treatment at a large Australian tertiary cancer care centre**

Georgia M. Lester, Wayne J. Wilson, Barbra H. B. Timmer and Rahul Ladwa

Corresponding author email: [georgia.lester@uq.edu.au](mailto:georgia.lester@uq.edu.au).

Supplementary material 2: List of primary site name and patient count

| Diagnosis Primary Site | Count |
| --- | --- |
| Malignant neoplasm tonsil unspecified | 26 |
| Malignant neoplasm of base of tongue | 18 |
| Malignant neoplasm upper lobe bronchus or lung | 15 |
| Malignant neoplasm nasopharynx unspecified | 9 |
| Malignant neoplasm of testis unspecified | 6 |
| Malignant neoplasm of supraglottis | 6 |
| Malignant neoplasm long bones lower limb | 5 |
| Malignant neoplasm lower lobe bronchus or lung | 4 |
| Malignant neoplasm oropharynx unspecified | 3 |
| Malignant neoplasm of thymus | 3 |
| Malignant neoplasm of tonsillar fossa | 3 |
| Malignant neoplasm primary site unspecified | 3 |
| Malignant neoplasm of gallbladder | 3 |
| Malignant neoplasm lower third oesophagus | 3 |
| Malignant neoplasm extrahepatic bile duct | 2 |
| Malignant neoplasm lat wall nasopharynx | 2 |
| Intrahepatic bile duct carcinoma | 2 |
| Mesothelioma of pleura | 2 |
| Diffuse large B-cell lymphoma | 2 |
| Malignant neoplasm of ethmoidal sinus | 2 |
| Malignant neoplasm of bladder neck | 1 |
| Malignant neoplasm tongue unspecified | 1 |
| Malignant neoplasm lat floor of mouth | 1 |
| Malignant neoplasm of bladder unsp | 1 |
| Malg neoplm con & soft tis low limb hip | 1 |
| Malignant neoplasm of body of stomach | 1 |
| Malignant neoplasm of undescended testis | 1 |
| Malignant neoplasm of border of tongue | 1 |
| Malignant neoplasm post wall nasopharynx | 1 |
| Malignant neoplasm of descended testis | 1 |
| Malg neoplasm upp inner quadrant breast | 1 |
| Malg neoplm con / soft tis upp lmb shold | 1 |
| Overlap malg lesion of bronchus & lung | 1 |
| Malg neoplasm ant tongue part unsp | 1 |
| Malignant neoplasm laryngeal cartilage | 1 |
| Malignant neoplasm of glottis | 1 |
| Malignant neoplasm of vallecula | 1 |
| Malignant neoplasm of nasal cavity | 1 |
| Malignant neoplasm post wall hypopharynx | 1 |
| Malignant neoplasm of pyriform sinus | 1 |
| Malg neoplasm of posterior wall bladder | 1 |
| Malignant neoplasm of retromolar area | 1 |
| Malg neoplasm pelvic bones sacrum coccyx | 1 |
| Malignant neoplasm of soft palate | 1 |
| Mesothelioma of peritoneum | 1 |
| Malignant neoplasm of subglottis | 1 |
| Nodular lymphocyte predom Hodgkin lymph | 1 |
| Extranodal NK/T-cell lymphoma nasal type | 1 |
| Unknown Primary site | 1 |
| Malignant neoplasm ant floor of mouth | 1 |
| Malignant neoplasm anterior wall bladder | 1 |
