# Supplementary material for: A retrospective audit of audiology encounters in patients undergoing Cisplatin treatment at a large Australian tertiary cancer care centre
Source: J Cancer Surviv. 2024 Oct 15;20(2):816–22. doi: 10.1007/s11764-024-01689-x (PMC12988975; doi:10.1007/s11764-024-01689-x)
Supplement: Supplementary file 3 — Supplementary file3 (DOCX 17 KB) [file 11764_2024_1689_MOESM3_ESM.docx]

**A retrospective audit of audiology encounters in patients undergoing Cisplatin treatment at a large Australian tertiary cancer care centre**

Georgia M. Lester, Wayne J. Wilson, Barbra H. B. Timmer and Rahul Ladwa

Corresponding author email: [georgia.lester@uq.edu.au](mailto:georgia.lester@uq.edu.au).

# Supplementary material 3: List of diagnoses morphology and patient count

| Diagnosis Morphology | Count |
| --- | --- |
| Squamous cell carcinoma NOS | 48 |
| Squamous cell carcinoma, large cell, nonkeratinising NOS | 25 |
| Squamous cell carcinoma, keratinising NOS | 12 |
| Adenocarcinoma NOS | 9 |
| Acinar cell carcinoma | 6 |
| Osteosarcoma NOS | 4 |
| Seminoma NOS | 4 |
| Carcinoma, undifferentiated NOS | 3 |
| Cholangiocarcinoma | 3 |
| Small cell carcinoma NOS | 3 |
| Basaloid squamous cell carcinoma | 2 |
| Transitional cell carcinoma NOS | 2 |
| Diffuse large B-cell lymphoma NOS | 2 |
| Mixed germ cell tumour | 2 |
| Combined small cell carcinoma | 1 |
| Giant cell sarcoma | 1 |
| Epithelioid mesothelioma, malignant | 1 |
| Infiltrating duct carcinoma NOS | 1 |
| Sarcoma NOS | 1 |
| Large cell neuroendocrine carcinoma | 1 |
| Carcinoma NOS | 1 |
| Mesothelioma, biphasic, malignant | 1 |
| Thymic carcinoma NOS | 1 |
| Mesothelioma, malignant | 1 |
| Hodgkin lymphoma, nodular lymphocyte predominant | 1 |
| Adenoid cystic carcinoma | 1 |
| Choriocarcinoma NOS | 1 |
| Neuroendocrine carcinoma NOS | 1 |
| Solid carcinoma NOS | 1 |
| NK/T-cell lymphoma, nasal and nasal-type | 1 |
| Squamous cell carcinoma, HPV positive | 1 |
| Adenocarcinoma with mixed subtypes | 1 |
| Fibroblastic osteosarcoma | 1 |
| Papillary adenocarcinoma NOS | 1 |
| Thymoma, type B2 | 1 |
| Papillary urothelial carcinoma | 1 |
| Yolk sac tumour | 1 |
| Parosteal osteosarcoma | 1 |
| Pleomorphic carcinoma | 1 |
